# Developmental conditions promote individual differentiation of endocrine axes and behavior in a tropical pinniped

**DOI:** 10.1007/s00442-020-04815-5

**Published:** 2020-12-19

**Authors:** Eugene J. DeRango, Jonas F. L. Schwarz, Friederike Zenth, Paolo Piedrahita, Diego Páez-Rosas, Daniel E. Crocker, Oliver Krüger

**Affiliations:** 1 Department of Animal Behaviour, Bielefeld University, Morgenbreede 45, 33615 Bielefeld, Germany; 2 University of Freiburg, Freiburg, Germany; 3 Facultad de Ciencias de la Vida, Escuela Superior Politécnica del Litoral, Guayaquil, Ecuador; 4 Galápagos Science Center, Universidad San Francisco de Quito, Isla San Cristόbal, Galápagos, Ecuador; 5 Dirección Parque Nacional Galápagos, San Cristóbal, Isla San Cristóbal, Galápagos, Ecuador; 6 Department of Biology, Sonoma State University, Rohnert Park, CA USA

**Keywords:** Galapagos sea lion, Hormones, Maternal effects, Metabolism, Personality

## Abstract

Between-individual variation in behavior can emerge through complex interactions between state-related mechanisms, which include internal physiological constraints or feedback derived from the external environment. State-related conditions can be especially influential during early life, when parental effort and exposure to social stress may canalize consistent differences in offspring hormonal profiles and foster specific behavioral strategies. Here, we unravel how relevant state variables, including sex, somatic condition, local population density, and maternal traits, contribute to within-cohort differences in stress, sex, and thyroid hormone axes in dependent Galapagos sea lions with the primary goal of understanding downstream effects on boldness, docility, habitat use, and activity. Pups within denser natal sites had higher levels of cortisol and thyroid T4, a prohormone and proxy for metabolic reserves, likely as an adaptive physiological response after exposure to increased numbers of conspecific interactions. Furthermore, considering maternal effects, mothers in better body condition produced pups with higher testosterone yet downregulated basal cortisol and thyroid T4. This hormonal profile was correlated with increased boldness toward novel objects and attenuated stress responsiveness during capture. Intriguingly, pups with increased thyroid T3, the biologically active form, maintained faster somatic growth and were observed to have increased activity and extensively explored surrounding habitats. Collectively, these findings provide comprehensive evidence for several links to hormone-mediated behavioral strategies, highlighted by variation in socio-environmental and maternally derived input during a foundational life stage.

**Supplementary Information:**

The online version contains supplementary material available at 10.1007/s00442-020-04815-5.

## Introduction

The concept that many aspects of behavior are highly variable across individuals within most species is now deeply rooted into modern ecological studies (Wolf et al. 2007). Although behavior can acutely change in response to environmental stimuli, individuals often exhibit consistent differences in average behavioral phenotypes over time or within and across contexts (deemed animal ‘personalities’) (Sih et al. 2004). Traits such as boldness (i.e., risk-taking), exploration, or sociability seem functionally distinct but are often linked through correlations known as behavioral syndromes (Réale et al. 2007). The structure of these traits may underlie how animals occupy specific niches within their habitat (Dingemanse and Wolf 2010; Wolf and Weissing 2010). For example, bold animals may utilize more risk-intense environments, which in turn could result in receiving higher rates of agonistic interactions or even predation. Although descriptions of between-individual variation in behavior are now plentiful for a variety of taxa, an outstanding question remains as to which proximate mechanisms are the key determinants in promoting these differences (Wilson et al. 2019). Recent theoretical models have put forth that behavioral phenotypes arise through complex interactions between any number of ‘states’, which can be summarized as intrinsic characteristics, such as morphology or internal physiology, or differential feedback received from the physical or social environment (Sih et al. 2015). Undoubtedly, state-dependent feedback on behavior is multi-faceted and tightly interwoven into the ecology of individuals and entire species (Luttbeg and Sih 2010); however, studies often isolate only one or few aspects of state-trait variation and may neglect possible synergistic relationships or overlap in variance between relevant states (Dingemanse et al. 2012; Niemelä and Dingemanse 2018). For example, could sex differences or an individual’s position within conspecific groups both contribute to risk-perception or novelty-seeking within a habitat? And to what degree are hormonal or metabolic differences underpinning these effects (Careau et al. 2008; Biro and Stamps 2010)? Therefore, an integrated approach is needed to unify environmental and physiological state variables and ultimately decipher which promote behavioral differentiation within naturally complex wildlife systems (Dammhahn et al. 2018; Salzman et al. 2018).

Hormonal profiles are important foundations for understanding how state-dependent variation acts on behavioral phenotypes, as they are highly dependent on internal and environmental feedback and can accurately reflect the current state of an individual (Romero et al. 2009; Dantzer et al. 2016). Glucocorticoids (predominantly cortisol in mammals) often receive focus because they are chemical messengers with powerful stimulatory or prohibitive effects to redirect energetic reserves and appropriately mediate behavior (Sapolsky et al. 2000; Jimeno et al. 2018). This is especially relevant considering that energy is a major constraint on all animals when engaging in specific behaviors (Careau et al. 2008; Biro and Stamps 2010). Consistent (i.e. repeatable) patterns of basal hormone levels often emerge, which can be inherited or influenced by a host of intrinsic or extrinsic states, such as current energetic constraints (Vitousek et al. 2014; Taff et al. 2018). When coupled with an understanding of personality traits, the strength and consistency of hormonal responses can yield insights into how individuals emphasize certain behaviors when responding to environmental cues (Hau and Goymann 2015). For example, because glucocorticoids are directly involved in coping with unpredictable or challenging events, such as exposure to unfamiliar situations, they have been extensively documented to regulate risk-taking, exploration behavior, and patterns of stress reactivity (Coppens et al. 2010; Koolhaas et al. 2010).

Furthermore, because of the complexities of the neuroendocrine system, researchers have also called for an improved understanding of interactions between multiple endocrine axes and their effects on individual behavior (Sih et al. 2015; Wilson et al. 2019). For example, sustained high levels of cortisol can concurrently alter the production of reproductive hormones like testosterone (Castañeda et al. 2014) or stimulate the thyroid axis to increase basal metabolic rates (McNabb and King 1993). This can occur when cortisol promotes the conversion of thyroxine (T4, a reservoir prohormone) into the biologically active triiodothyronine (or T3), which carries out cellular metabolism and promotes somatic growth (Charmandari et al. 2005). While testosterone is well-documented to be sex-specific and correlate with dominance, status-seeking, or aggressive behavior towards conspecifics (Wingfield et al. 1990; Mehta and Josephs 2010), thyroid hormones currently receive the little spotlight in ecological studies but show promise in predicting patterns of overall activity and metabolic capacities (Helmreich and Tylee 2011; Cristόbal-Azkarate et al. 2016). Thus, quantifying hormone axes as a representation of physiological state variables should clarify these overarching patterns and allow us to postulate how individuals face challenges within life history contexts (Wolf and McNamara 2012; Holtmann et al. 2017).

Of all life stages, early development reflects a period in which state conditions have some of the strongest effects to foster intrinsic differences in hormonal and behavioral phenotypes (Stamps and Groothuis 2010; Sih 2011; Trillmich et al. 2018). During this time, offspring are shaped by a suite of ontogenetic processes (Badyaev and Uller 2009), each of which could be deleterious or adaptive depending on pressures within the early life environment. Parental effects have obvious and powerful influences on resource allocation towards dependent offspring (Reddon 2012). Experimental studies have broadly shown that maternally derived input regulates offspring body condition and differential hormone exposure, which concurrently later affect available energetic reserves for costly behaviors (Del Giudice 2012). For gregarious species, offspring are also quickly exposed to dynamic interactions within the social environment. As seen in rodent models, the quality and quantity of social cues create huge fluctuations in energetic demands, which over time may canalize hormonal and behavioral phenotypes to cope with these requirements (Kaiser et al. 2003; Sachser et al. 2011). Accordingly, understanding interactions between early life states are critical in framing how these conditions may steer offspring towards consistent differences in behavioral phenotypes.

A long-term study of Galapagos sea lions (GSL, *Zalophus wollebaeki*) provides an excellent avenue to untangle a host of developmental and state-dependent effects on behavioral differentiation in a unique wildlife population. As GSL show island tameness and are easily approachable, this species has allowed for a rare glimpse into describing intraspecific variation in personality traits within this taxon (DeRango et al. 2019a, b). These studies of dependent pups revealed strong between-individual consistency in boldness (mean repeatability estimate *R* = 0.70 ± 0.06 SE), and that boldness is linked to variability in stress coping responses (i.e. docility). However, the physiological mechanisms which drive these traits during early life currently remain unknown, but likely involves some combination of maternal and environmentally derived feedback. Like all otariids (eared seals), adult female GSL are income breeders, meaning they continuously forage at sea and return to terrestrial aggregations to nurse and invest in a single altricial pup. As a result, demands on GSL mothers are extremely high to provide energy to offspring over an extensive dependency period (Piedrahita et al. 2014; Trillmich et al. 2014; Páez-Rosas and Guevara 2017). For many pinnipeds, maternal age and body condition have been shown to impact driving ability, foraging success, and accordingly, energetic investment towards pups (Hassrick et al. 2013; Hooper et al. 2019). Furthermore, immature GSL shares diverse natal sites that facilitate varying degrees of social competition and agonistic behavior (Wolf and Trillmich 2007), which along with maternal effort, are hypothesized to be linked to pronounced variation in offspring endocrine and behavioral phenotypes (Meise et al. 2016).

In this study, we explore how several state-dependent variables could contribute to between-individual hormonal variation in dependent pups, and in turn how this may differentially influence within-cohort variation in a suite of measured behaviors. To test these relationships, our plan was threefold. We first determined the repeatability of concentrations of near-baseline cortisol and testosterone during the perinatal period. We also followed individuals one year into the dependency period to further determine the stability of these hormones and covariation with serum thyroid hormones T4 and T3 as indicators of metabolic activity. Based on the state-dependent model, we hypothesized that between- and within-individual consistency would be needed to understand how differences in behavior may be maintained. We then drew on established methods to determine between-individual differences in boldness and docility, and, additionally here, calculate long-term assessments of activity level and spatial habitat movement through non-invasive observations. Finally, by combining these datasets, we examined whether state-dependent variables were related to the expression of hormones and established behaviors. For this, we used structural equation modeling to determine the strength of a priori mechanistic pathways. Specifically, we hypothesized that pup sex and body condition, socio-environmental context (i.e. local population density), and maternal state variables (age and body condition) would directly contribute to hormonal profiles of focal pups, leading to downstream effects on behavioral patterns.

## Materials and methods

### Study details

**Fig. 1:**
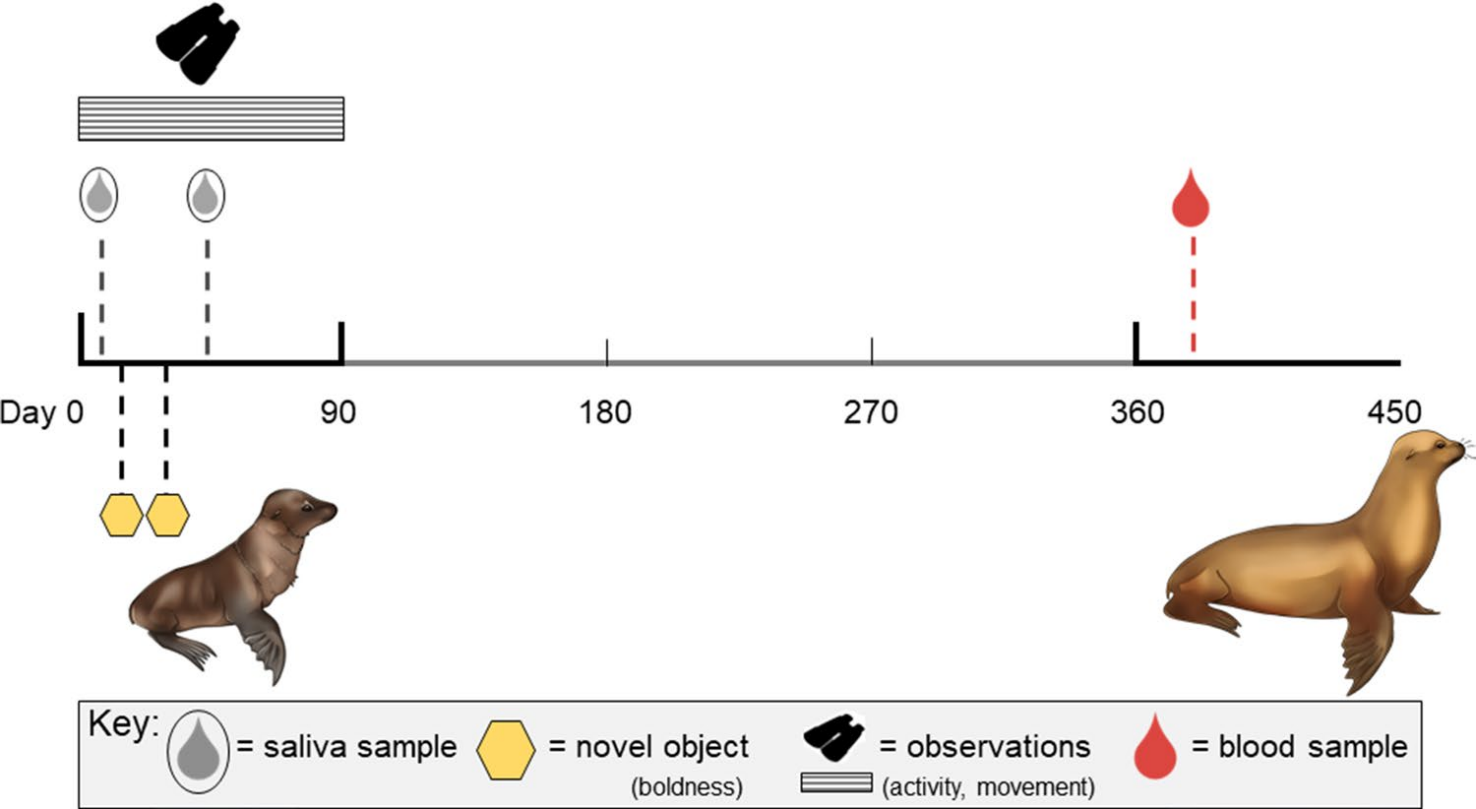
Schematic of behavioral and hormonal data collection. Focal sea lion pups (*N* = 86) underwent behavior assessments and handling events between 0–90 days old, and a final capture as yearlings. Dashed vertical lines represent mean days old for all individuals during each event, and the horizontal bar represents the duration of behavioral observations

Our dataset stems from extensive monitoring of an important breeding colony in the central region of the Galápagos archipelago. Since 2003, the islet Caamaño (0°45′S, 90°16′W) has been mapped to effectively gather individual-based demographic and life history information on resident sea lions (Trillmich et al. 2016). Caamaño is ~ 300 m in diameter and varies greatly in habitat suitability based on the topography (Wolf and Trillmich 2007). The peak timing of parturition, and therefore annual field seasons, extends between October and December. Specific to this study, we collated fine-scale individual behavioral data from focal pups during the reproductive seasons of 2017–2019 (2017, *N* = 41; 2018, *N* = 29; 2019, *N* = 16) Using longitudinal data, we determined the age of known mothers for most pups (*N* = 69, mean = 12.2 ± 3.5 years SD). Between a standardized period of 0–90 days after birth, focal pups undergo daily behavioral observations and novel object tests (detailed below). Starting 2–6 days postpartum (when mothers leave for foraging trips), pups also undergo a routine handling regime (mean captures = 3 ± 1 SD per individual). During captures, we collect saliva samples, apply unique numerical shaves for identification, confirm sex, and measure standard body length and mass. Individuals are followed as yearlings during the subsequent season to place permanent tags, obtain morphometrics, and collect blood samples for hormone analysis. The timeline of these events is detailed in Fig. 1. Morphometric traits are later used to calculate a scaled mass index (SMI, Peig and Green 2009), or body condition metric which considers allometric scaling. Specifically, here, we used a rate of change, or Δ SMI, rather than a static timepoint to understand the overall maintenance of body condition during the first year of life. We also calculated maternal SMI using morphometrics from a single capture for adult females, as part of a separate diving physiology study.

### Focal behaviors

We adopted methods previously used in DeRango et al. (2019b) to determine boldness and docility scores for all individuals. Boldness was determined using responses to a novel object test previously designed for use in young sea lions. These tests were repeated twice during the 0–90 period, spaced approximately 2 weeks apart. Mean durations of behaviors during the trials were entered into a principle component analysis (DeRango et al. 2019b), and here we focus on PC1 defined as boldness. PC1 was time spent in proximity of the test area and latency to flee when presented with the novel object (as shown in Online Resource 1). Because some but not all individuals showed adverse reactions, we considered this to be a risk-taking behavior.

Docility was characterized as the degree of resistance to manual restraint (i.e. struggle) across multiple capture events (DeRango et al. 2019b). Scores were measured along an ordinal rank scale, from no to severe struggle. Although these scores were not found to be highly repeatable across multiple time points (mean repeatability estimate *R* = 0.222 ± 0.110), we found personality-dependent effects of the plasticity of these responses over time. Rather than intercepts or fixed points, here we created a ‘docility score’ by using individual slopes from mixed model ordinal regressions, previously calculated using struggle scores as dependent effects and time in days as a fixed effect (DeRango et al. 2019b). This slope value was multiplied by − 1; thus, higher positive docility values indicate faster habituation during the 0–90-day period.

New to this study are measures of activity level, movement behavior, and local population density. These metrics were calculated simultaneously during 3 daily census surveys wherein we recorded focal behaviors, current locations, and nearby conspecifics. Arnold and Trillmich (1985) was used as a guideline for activity budgets, determined as a percentage of behaviors categorized as active vs. inactive. Behavior was recorded using instantaneous scan sampling within a 10-s window. Active behavioral states were defined as (1) general interaction or play with other sea lions, heterospecifics or inanimate objects; (2) locomotion not involving play, and (3) grooming, i.e*.* scratching the body. Inactive behaviors were (1) lying or sleeping in a prone position; or (2) sitting upright without locomotion. Behaviors were mutually exclusive, with only the more active category recorded per observation. For movement patterns, we noted in which site a pup was found and calculated the total number of movements between sites as an indicator of habitat exploration (see DeRango et al. 2019b for site divisions). Local density was individual-specific by noting conspecifics within a 3-body length radius of the focal animal. All metrics were summed and divided by the number of observations recorded per individual to determine rates for behaviors and mean neighbors present per observation.

### Sample collection for hormone analyses

We developed a novel field protocol to collect saliva during the first and last captures for each pup, separated by a mean of 37 days ± 12 SD. Two cotton swabs were rotated for 30 s each in the cheek pouch and under the tongue. We reliably collected ~ 150 µl of saliva between 15 s and 2 min post-disturbance, which represents appropriate time frames to measure basal hormone values (Bozovic et al. 2013). Samples were processed to quantify free, unbound CORT, and TEST (hereafter CORT_sal_ and TEST_sal_).

A subset of individuals born in 2017 (*N* = 30) and 2018 (*N* = 27) were captured again as yearlings (mean age ± SD = 381 ± 44 days old). Due to safety concerns with larger animals, we opted to use blood rather than saliva for serum analysis of total CORT and TEST (hereafter CORT_ser_ and TEST_ser_) to correlate these with saliva measurements in addition to measure thyroid hormones T3 and T4. Yearlings were restrained within modified hoop nets in a prone position (Fuhrman Diversified Inc., Texas, USA), and blood was collected within 3 min from the caudal gluteal vein to similarly reflect basal hormones (Romero and Reed 2005).

All hormones were quantified in duplicate using commercially available assay platforms. CORT_sal_ and TEST_sal_ were measured using extended range enzyme immunoassays (EIA) from Salimetrics, LLC, USA. CORT_ser_, total T3 (TT3_ser_), and total T4 (TT4_ser_) were measured using a hormone specific I^125^ RIA coated tube kit, while TEST_ser_ was measured using an EIA kit from MPBiomedicals (MP Biomedicals, Orangeburg, NY, USA). Sample processing and hormone quantification, including recovery and parallelism validations, are detailed in Online Resource 2.

### Statistical analyses

We first tested the temporal consistency of CORT_sal_ and TEST_sal_ by calculating population-level repeatability estimates, or R, by using the *rptR* package (Stoffel et al. 2017) in R (version 3.5.1). Specifically, the rptGaussian function was applied after log transforming all hormone values to achieve normality and meet model assumptions. We used parametric bootstrapping to recreate 1,000 iterations and deemed estimates as significant if *P* < 0.05. We then used R package *lme4* (Bates et al. 2014) to create linear mixed models (LMMs) and manually extract individual variance to determine an adjusted metric of within-individual level repeatability or *R*_*i*_. This was calculated as the between-individual variance divided by the sum of the between-individual plus residual variance for each individual animal (Nakagawa and Schielzeth 2010; Dingemanse and Dochtermann 2013). Both estimates are scaled from 0 to 1, from slight or low (0.0–0.4) to high (0.7 +) repeatability (Harper 1994). We applied another LMM to compare CORT_sal_ and TEST_sal_ at 0–90 days old with serum measurements at 1 year. CORT_sal_ and TEST_sal_ were inputted as predictor variables, individual ID as a random effect, and CORT_ser_ and TEST_ser_ as response variables. Furthermore, to affirm that our measurements reflect basal values, time after disturbance (in min) was accounted for but subsequently removed due to lack of significance and ability to explain a significant amount of variance.

**Fig. 2:**
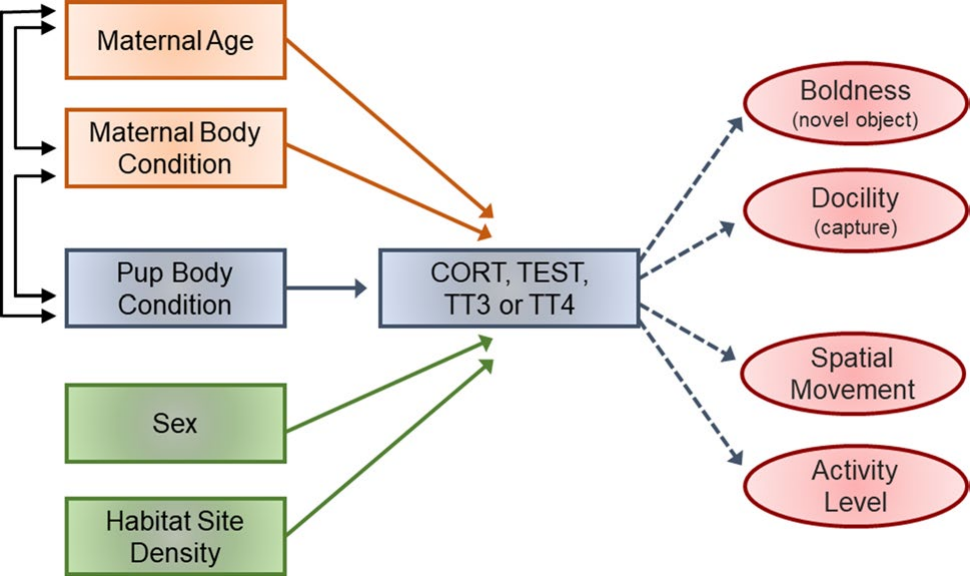
Hypothesized pathways used to determine state-related feedback on individual behavioral variation. Four separate structural equation models were set up for each hormone. Sea lion illustrations credit: Janina Weissenborn. The color version of the figure is available online

We then created structural equation models (i.e. path analyses) using AMOS version 21.0 (Chicago: IBM SPSS) to test our hypothesized connections between all available state variables and behaviors (Fig. 2). Path analyses are generally recommended, as they standardize variable estimates and can identify multi-directional associations between endocrine, behavioral, or life-history traits while weighing the effects of intercorrelated variables in the model (Dingemanse et al. 2010; Dantzer et al. 2016). Four separate models were created for each respective hormone (CORT_ser_, TEST_ser_, TT3_ser_, TT4_ser_). We chose to enter serum hormones into the path analyses rather than saliva, as this allowed for the greatest number of comparisons between endocrine axes within a specific sample type. Pup sex (coded as females = 1, males = 2), Δ SMI, local density, maternal age, and SMI were the state-dependent effects entered as exogenous variables (i.e. not influenced by other variables). The hormone in question and each behavior were considered endogenous (i.e. influenced by variables pointing to them). Within each model, we also included covariation between pup sex, Δ SMI, and both maternal traits, as these variables are likely inextricably linked. Maximum likelihood was used to calculate standardized regression estimates, factor score weights, confidence ratios, and p-values for each path, with alpha values for path relationships set at *p* < 0.05 and approached significance when *p* < 0.10. Goodness of fit was confirmed through appropriate model Chi-squared values. Because AMOS does not allow for assessment of covariation between endogenous variables, we separately determined within-individual correlations between all serum hormones according to multivariate Pearson’s correlations prior to model fitting. The datasets generated during and/or analyzed during the current study are available in the Mendeley Data repository, http://dx.doi.org/10.17632/8cn4g6ppxx.1.

## Results

### Individual hormone stability and covariation

Between-individual repeatability estimates for basal CORT_sal_ TEST_sal_ were moderately high to high (*R* ± SE = 0.685 ± 0.127 and 0.849 ± 0.165, respectively) across both capture events during the perinatal period. Within-individual repeatability was also high across most animals (CORT_sal_ *R*_*i*_ ± SE = 0.742 ± 0.266, T_sal_ *R*_*i*_ ± SE = 0.631 ± 0.260). When the subset of individuals were recaptured as yearlings, we found that salivary CORT and TEST were positively correlated with serum measurements (LMM: mean CORT_sal_:CORT_ser_ association—*R*^*2*^ = 0.44, *p* = 0.001; mean T_sal_:T_ser_ association—*R*^*2*^ = 0.40, *P* = 0.042), indicating that hormonal profiles across both sample types mostly remained stable within individuals during the first year of dependency. Furthermore, considering covariation between endocrine axes, we found negative correlations between CORT_ser_ and TEST_ser_ (Pearson’s *r* = *− *0.47, *P* = 0.014), and separately between thyroid TT4_ser_ and TT3_ser_ (Pearson’s *r* = − 0.17, *P* = 0.020), but no correlations between CORT_ser_ or either thyroid hormone (Pearson’s *r, P* > 0.05).

### Pathways affecting endocrine and behavioral variation

**Fig. 3:**
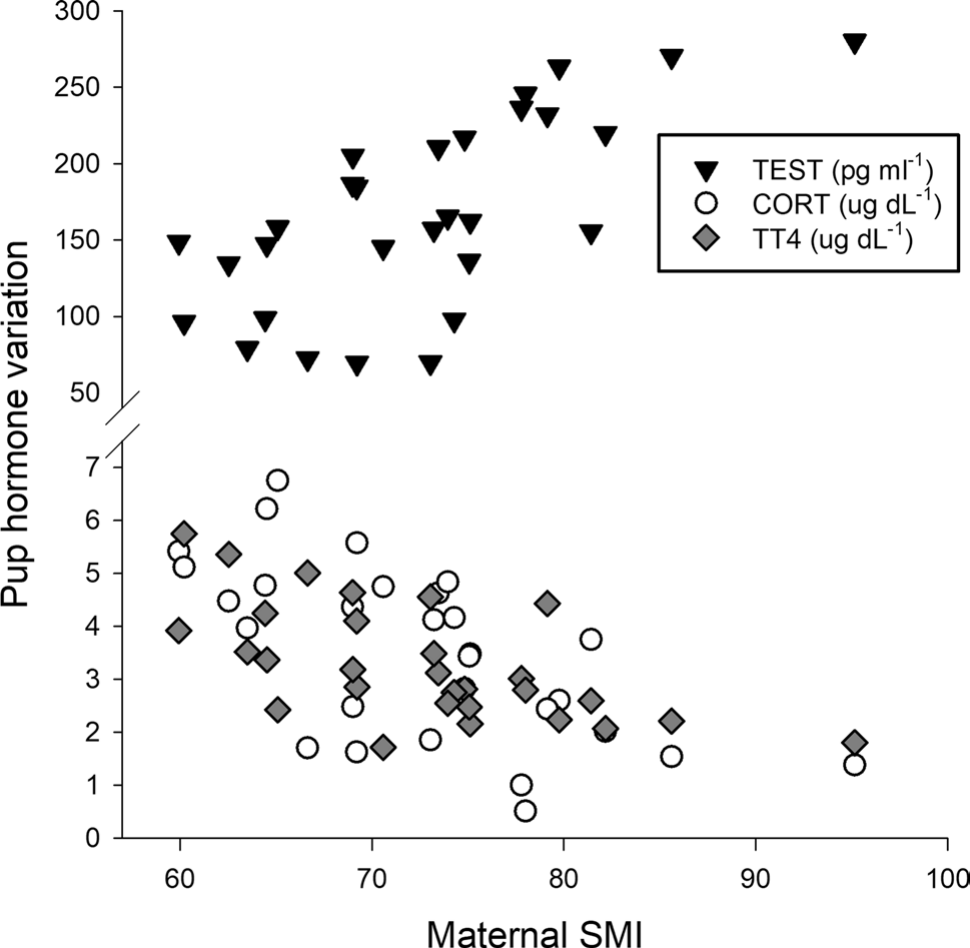
Raw linear plots showing significant relationships between maternal body condition (SMI, or scaled mass index) and variation in serum hormonal profiles for dependent pups. Hormone concentrations were standardized to fit the scaled axis

We found support for multiple direct exogenous effects contributing to each hormone pathway model (Table 1). Local population density affected CORT_ser_ and TT4_ser_ reservoirs similarly, wherein individuals experiencing greater exposure to conspecifics had higher basal levels of both hormones_._ Sex differences in testosterone were also already present, with male pups having higher TEST_ser._ Considering maternal effects, only maternal SMI had a strong influence on pup hormone profiles; females in better body condition produced pups with lower CORT_ser_ and TT4_ser_ but higher TEST_ser_ (Fig. 3). Although there was no direct covariation between any maternal traits and pup Δ SMI (Table 2), pups with faster relative increases in body condition (i.e. greater Δ SMI) had higher CORT_ser_ and TT3_ser_ values.

**Table 1:** Path coefficients (path coef.) derived from 4 different structural equation models where variation in hormones were examined separately

Pathway	CORT_ser_		TEST_ser_		TT4_ser_		TT3_ser_	
	Path Coef	*P*	Path Coef	*P*	Path Coef	*P*	Path Coef	*P*
Local density →	**0.312**	**0.034**	0.006	0.96	**0.276**	**0.034**	0.157	0.31
Sex →	− 0.169	0.26	**0.494**	**< 0.001**	0.001	0.99	0.087	0.58
Δ SMI →	**− 0.323**	**0.037**	0.176	0.19	**− 0.378**	**0.006**	**0.434**	**0.008**
Matern. age →	0.086	0.57	0.180	0.18	− 0.146	0.35	0.053	0.75
Matern. SMI →	**− 0.423**	**0.005**	**0.525**	**< 0.001**	**− 0.575**	**< 0.001**	0.196	0.22

	Boldness		Docility		Movement		Activity level	
	Path Coef	*P*	Path Coef	*P*	Path Coef	*P*	Path Coef	*P*
CORT_ser_ →	**− 0.440**	**0.007**	− 0.167	0.35	0.183	0.31	0.232	0.19
TEST_ser_ →	**0.676**	** < 0.001**	**0.346**	**0.045**	− 0.078	0.67	− 0.259	0.14
TT4_ser_ →	− 0.236	0.18	**− 0.425**	**0.010**	− 0.102	0.58	0.159	0.38
TT3_ser_ →	− 0.084	0.64	**− 0.507**	**0.001**	**0.380**	**0.024**	.296	0.091^T^

Exogenous variables which link directly to each hormone are positioned above, while endogenous hormone levels linked to behavioral traits are positioned below. Significant path coefficients (*P* < 0.05) are in bold, while ^T^ denotes those that approach significance (*P* < 0.10)

**Table 2:** Covariation between exogenous variables within each structural equation model

Covariance	Estimate	S.E	C.R	*P*
Maternal age ← → Maternal SMI	− 0.163	5.584	− 0.881	0.38
Maternal age ← → Pup Δ SMI	0.270	1.079	1.44	0.15
Maternal SMI ← → Pup Δ SMI	**− **0.151	2.682	− 0.828	0.41
Pup Δ SMI ← → Pup sex	− 0.151	0.142	− 0.853	0.39

Weighted covariance estimates, standard error (S.E.), critical ratios (C.R.), and *p*-values are given

**Fig. 4:**
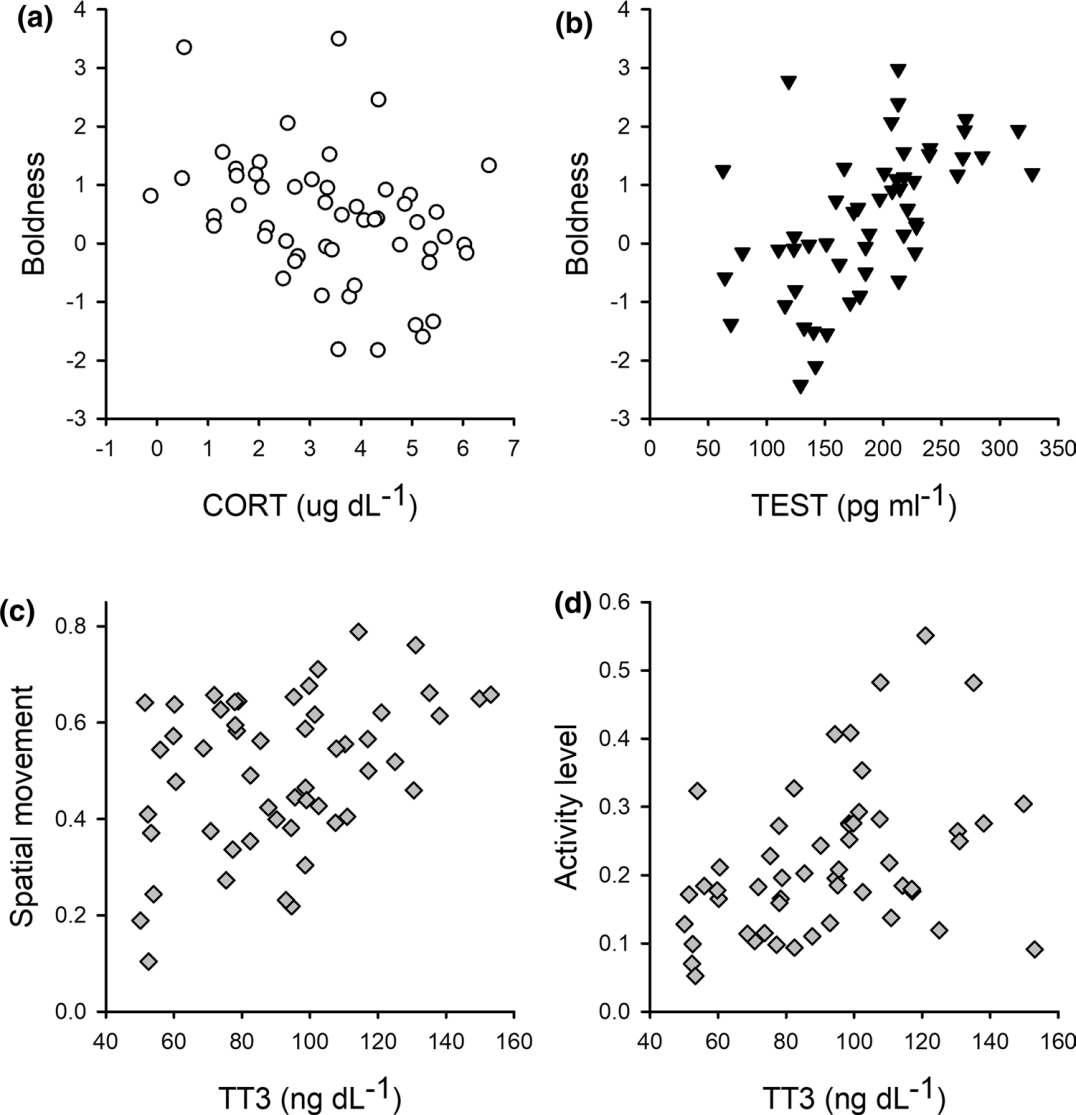
Raw linear plots showing significant relationships between serum cortisol (CORT, **a**) and testosterone (TEST, **b**) on boldness, and between thyroid hormone T3 on movement (**c**) and activity level (**d**) rates

Several significant pathways also existed between endogenous hormone variables and behavioral traits (Table 1). Only CORT_ser_ was associated with boldness towards novel object presentation and was lower in those that were bolder (i.e. showed a reduced latency to flee and were less neophobic) (Fig. 4). Individuals with higher TEST_ser_ yet lower levels of TT4_ser_ and TT3_ser_ were more docile, with attenuated stress responses towards capture. Furthermore, higher TT3_ser_ was associated with higher rates of movement between habitat sites and a tendency towards increased activity level (i.e. general locomotion and conspecific interaction).

## Discussion

In wildlife populations, identifying relevant state-dependent conditions is fundamental towards understanding behavioral variation at the individual and population level (Wolf and Weissing 2010; Sih et al. 2015). The early postnatal period for Galapagos sea lions provides several state variables to consider which we demonstrate to alter hormonal and behavioral phenotypes, including local population densities and maternal condition. Further, we found strong variability in hormone axes, specifically near-baseline levels of CORT, testosterone, and thyroid hormones, within an exceedingly complex developmental window. This is exceptionally important to the foundation of this study because these metrics reflect large between-individual variance and within-individual consistency in pup hormone levels within this context, thus allowing us to postulate about their ecological relevance and effects on individual personalities at this early stage.

### Socio-ecological states

Physical conditions on Caamaño have been shown to create variability in the degree of exposure to conspecifics within the early life environment (Wolf and Trillmich 2007). Here, pups that experienced more exposure to conspecifics during the perinatal period had consistently higher levels of CORT. These results mirror a study of terrestrial habitat quality in Antarctic fur seals (*Arctocephalus gazella*), wherein adult females are exposed to a high density of neighboring conspecifics, and therefore agonistic interactions and social stress, also had upregulated CORT levels (Meise et al. 2016). Although we were unable to specifically measure the repeatability of thyroid hormones, TT4 in tandem with CORT was also higher in this context. These patterns likely affirm that dense environments acutely require higher hormone levels and basal metabolic rates to upregulate behavior, as seen in social rodents (Sachser et al. 2011; von Engelhardt et al. 2015). It should be noted that, according to a study of the population structure of habitat sites on Caamaño, higher local site densities are also synonymous with preferred habitat (e.g. easy access to tidepools and shade) and reflect optimal breeding and nursing conditions (Wolf and Trillmich 2007). From a life history perspective, these sea lions show strong site fidelity and often remain in natal sites throughout life (Wolf and Trillmich 2007). A study of wild arctic ground squirrels (*Urocitellus parryii*) found that denser environments continually increased metabolic demands and caused an upregulation of TT4 reservoirs and active TT3 across several life stages, leading to consistently high activity levels to be competitive within foraging and reproductive contexts (Wilsterman et al. 2015). Thus, continued monitoring of focal individuals in our study system could confirm the extent of the stability of hormone levels later in life and elucidate how the social environment could produce long-lasting energetic trade-offs outside of our study window.

Cortisol, testosterone, and thyroid hormones had varying degrees of influence on behaviors related to risk-taking and stress responsiveness. Although less pronounced in GSL, eared seal species are classic examples of marked behavioral and size dimorphism driven by intense intra-sex competition, even at a young age (Kruger et al. 2014; Piedrahita et al. 2014). Therefore, it was unsurprising to find early sex-specific biases towards higher testosterone in males, as testosterone is a key regulator of aggression and dominance towards conspecifics in reproductive contexts (Wingfield et al. 1990; Mehta and Josephs 2010). Further, CORT and testosterone were inversely related, likely due to the antagonistic effects of CORT on testosterone (Viau et al. 2001), and individuals with lower CORT and higher testosterone were bolder. In a study of wild male Barbary macaques (*Macaca sylvanus*), individuals with consistently lower GC activity were considered more ‘excitable’ (Tkaczynski et al. 2019), although this was defined as aggressive interactions and should not be equated with boldness. Nevertheless, studies such as these support that steroid hormones likely regulate behaviors related to risk-taking across many scenarios (Koolhaas et al. 2010; Sih 2011). Intriguingly though, in our study, basal CORT levels did not directly reflect changes in docility. This lack of a detectable correlation could be due to our use of near-baseline CORT rather than acute, short-term increases in CORT, which are more often implicated as regulating behavioral reactivity towards stressful events (Coppens et al. 2010; Koolhaas et al. 2010). Instead, TT4 and TT3 were higher in individuals which were more docile, or increased struggle during captures over time. If we assume, from a mechanistic standpoint, that struggle is energetically expensive, it is reasonable that reactive strategies such as these could be attributed to the permissive effects of higher metabolic rates within certain individuals. However, because high TT4 was apparent in high-density environments, it becomes difficult to decouple whether reduced docility is a direct product of hormonal differences or is related to sensitivity due to continuous exposure to stressors in these habitat types. Nevertheless, these relationships show an important distinction in how hormones differentially regulate risk-taking and stress responsiveness.

We show utility in using thyroid TT3 as a physiological correlate for somatic condition and reliable measurement of ‘performance’, here observed via activity and movement rates. Pups that improved in body condition faster had elevated TT3, likely by converting TT4 reservoirs (which showed an inverse relationship with Δ SMI). These trends agree with previous studies which show that developing pups upregulate TT3 levels when metabolizing lipids during natural fasts to be used towards somatic maintenance and growth, as seen in Australian (*Arctocephalus pusillus*) and subantarctic (*Arctocephalus tropicalis*) fur seals (Atkinson et al. 2011; Verrier et al. 2012). Collectively, body condition and circulating TT3 are representative of available energetic reserves, which should fuel consistent differences in activity rates and movement patterns (Campos-Candela et al. 2019). In one example, a study of juvenile cavies (*Cavia aperea*) found basal metabolic rates seemed to underlie an animal’s propensity to explore habitats and likely facilitated finding scarce resources (Guenther et al. 2014). Considering other motivations, it was shown in highly social, wild house finches (*Haemorhous mexicanus*) that reactive individuals were more flexible and ‘explorative’ in their behavior because they had the necessary energy to seek out intraspecific interactions in unfamiliar habitats (Moyers et al. 2018), although these studies used glucocorticoids to infer energetic capabilities. Similarly, here, thyroid and body size differences may underlie behavioral and social motivations for GSL pups to locomote more and interact with conspecifics in greater portions of their habitat. Furthermore, in human and rat models, the thyroid also correlates with the activity of central dopamine and serotonin systems (Strawn et al. 2004, Helmreich and Tyree 2011), which are responsible for sensation and novelty-seeking. Thus, pending further research, our results could inform how internal states also regulate exposure to environmental feedback, which may in turn foster social niches among individuals (Schirmer et al. 2019).

### Maternal effects on offspring hormone profiles

Considering the role of maternal investment, maternal age unexpectedly did not covary with the maintenance of pup body condition nor did it contribute towards hormonal differences between individuals. This is puzzling, as life-history theory predicts that older females should put more emphasis, or resources, on reproduction because physiological trade-offs and risks are generally smaller later in life (Wolf et al. 2007; McDonald et al. 2020). However, because GSL thrives in a tropical habitat, females continually cope with trade-offs related to unpredictable shifts in prey availability in addition to reduced seasonality and productivity (Mueller et al. 2011; Trillmich et al. 2014). Therefore, females optimize fitness by keeping reproductive effort low but stable throughout their lifetimes (Kalberer et al. 2018), which could mask any age-specific patterns that affect pup condition and energetic reserves.

Strikingly, however, maternal body condition within the study period strongly contributed to variation in pup hormonal profiles. For adult female pinnipeds, body condition is often indicative of acute differences in foraging and lactation efficiency within the current environmental landscape (Crocker and McDonald 2016). Specific to Galapagos sea lions, adult females show unusually high diversity in intraspecific foraging strategies with associated metabolic costs and dietary specializations (Villegas-Amtmann et al. 2008, 2017; Paez-Rosas et al. 2017), all of which may underscore body condition differences found here. In controlled studies, offspring often have marked increases in testosterone and masculinization of traits when the maternal diet is optimal during gestation, potentially because of the high metabolic demands required to facilitate androgens in utero (Rosenfeld and Roberts 2004; Pike and Petrie 2005). Therefore, larger mothers may be equipped to meet the energetic demands of producing pups with increased testosterone, and supply pups with higher T4 as a reservoir for metabolic output, which we show to manifest as behavioral differences. If costs are indeed higher, this could imply a direct fitness consequence and be one mechanism for which natural selection acts on pups with these hormonal profiles. These results are enticing and could have profound implications for understanding how offspring may inherit phenotypes via maternally derived input.

## Conclusion

In summary, we point to a suite of state-conditions which exert effects on hormone-mediated behavioral variation in this marine predator. External feedback from the early social environment (i.e. conspecifics within natal sites) and somatic condition for pups and mothers provided some of the strongest influences on hormonal profiles, which we further showed to have multi-directional effects on boldness, docility, and movement and activity patterns. These findings highlight hormonal and behavioral correlations and mechanistic linkages which could exist as syndromes in young animals. Because each of the aforementioned state-conditions hint at short and long-term fitness consequences and ecological pressures (Réale et al. 2010; Dammhahn et al. 2018), data such as these build a foundation to later understand life-history variation in this and other long-lived species.

## Supplementary Information

Below is the link to the electronic supplementary material.

Supplementary file1 (PDF 587 KB)

Supplementary file2 (MP4 18141 KB)
