# Supplementary material for: Developmental conditions promote individual differentiation of endocrine axes and behavior in a tropical pinniped
Source: Oecologia. 2020 Dec 19;195(1):25–35. doi: 10.1007/s00442-020-04815-5 (PMC7882553; doi:10.1007/s00442-020-04815-5)

Online Resource 2

State-related conditions promote individual differentiation in endocrine axes and behavior: insights into the early development of a tropical pinniped

Oecologia

Eugene J. DeRango*, Jonas F.L. Schwarz, Friederike Zenth, Paolo Piedrahita, Diego Páez-Rosas, Daniel E. Crocker, Oliver Krüger

*Affiliation: Bielefeld University, email: eugene_joseph.derango@uni-bielefeld.de

Methods for Saliva and Serum Sample Processing

After collection, saliva samples were processed by placing cut tips of the cotton swabs into a 1.5 ml Eppendorf microcentrifuge tube with a hole in the bottom. This tube was placed into a 2ml microcentrifuge tube, which was then centrifuged at 3,000 RPM for 15 minutes allowing the separated saliva to fall within the 2 ml tube. Samples were stored at room temperature in the field for approximately 2 weeks until later frozen at -80 C. This timeframe has been shown to not cause degradation of these hormones in saliva (Chen et al. 1992, Garde and Hansen 2005). Before analysis, saliva samples were thawed, centrifuged for 5 minutes at 3,000 RPM to remove mucins and particulates, and ran according to manufacturer's specifications. Due to sample limitations, we pooled 200 µl of remaining saliva to perform validations.

Blood samples were immediately centrifuged in the field at 3,000 RPM for 15 minutes to separate serum. Due to lack of a freezer in remote conditions and the instability of serum at room temperature, serum was diluted 1:1 via pipette with pure ethanol until stored at - 80°C. Before analysis, we centrifuged samples again to separate the ethanol supernatant containing dissolved hormones. Ethanol preserves lipophilic hormones within serum with minimal degradation over time (Goymann et al. 2007) and has been used successfully in similar conditions in previous studies with pinniped serum (DeRango et al. 2019).

References

Chen Y-M, Cintrón NM, Whitson PA (1992) Long-term storage of salivary cortisol samples at room temperature

DeRango EJ, Greig DJ, Gálvez C, Norris TA, Barbosa L, Elorriaga-Verplancken FR, Crocker DE (2019) Response to capture stress involves multiple corticosteroids and is associated with serum thyroid hormone concentrations in Guadalupe fur seals (*Arctocephalus philippii townsendi*). Mar Mam Sci 35:72-92

Garde AH, Hansen ÅM (2005) Long-term stability of salivary cortisol. Scand J Clin Lab Invest 65:433–436

Goymann W, Schwabl I, Trappschuh M, Hau M (2007) Use of ethanol for preserving steroid and indoleamine hormones in bird plasma. Gen Comp Endocrinol 150:191–195

Supplementary Table 1 Mean recovery percentage from 3 added standards to a known sample for each assay platform validation, with mean intra-assay coefficient of variation (CV) for all samples run in duplicate. Initial values for all platforms were on the standard curve provided with manufacturer's standards, except for 8 individuals that were at the detectable limit for salivary CORT (0.012 µg dL^{-1}).

Hormone	Concentrations	Mean Recovery (% ± SD)	Mean Intra-assay CV (% ± SD)
$CORT_{sal}$	0.037, 0.111, 0.333 ng dL^{-1}	96.4 ± 3.1	4.38 ± 3.41
$TEST_{sal}$	15.4, 38.4, 96 pg ml^{-1}	100.2 ± 2.6	3.71 ± 3.22
$CORT_{ser}$	1.0, 3.0, 10 µg dL^{-1}	99.7 ± 1.1	2.40 ± .48
$TEST_{ser}$	100, 500, 2000 pg ml^{-1}	97.3 ± 4.2	5.28 ± 2.36
$TT3_{ser}$	50, 100, 200 ng dL^{-1}	98.8 ± 2.9	3.28 ± 1.50
$TT4_{ser}$	2.0, 4.0, 8.0 µg dL^{-1}	100.6 ± 3.9	4.61 ± 1.29

Supplementary Table 2 Summary statistics for salivary and serum endocrine hormone concentrations derived from individual samples ran in duplicate

Analyte	*N*	Mean	SD	Range lower	Range upper
$CORT_{sal}$ (ng dL^{-1})	70	1.37	1.36	0.012	4.30
$TEST_{sal}$ (pg ml^{-1})	70	606.5	481.5	42.9	1303.5
$CORT_{ser}$ (µg dL^{-1})	57	3.37	1.80	0.738	6.89
$TEST_{ser}$ (pg ml^{-1})	57	183.3	71.9	78.5	355.9
Thyroid $T3_{ser}$ (ng dL^{-1})	57	92.7	31.5	42.2	164.2
Thyroid $T4_{ser}$ (µg dL^{-1})	57	3.29	1.26	1.67	6.17

Supplementary Fig. 1 Parallelism of mean absorbance units for EIA assays and % (B / B_0) values for RIA assay platforms. Serial sample dilutions with standard curves are shown for the following hormone concentrations: salivary cortisol ($CORT_{sal}$, A) and testosterone ($TEST_{sal}$, B), serum testosterone ($TEST_{ser}$, C), cortisol ($CORT_{ser}$, D), thyroid $T3_{ser}$ (E), and thyroid $T4_{ser}$ (F).

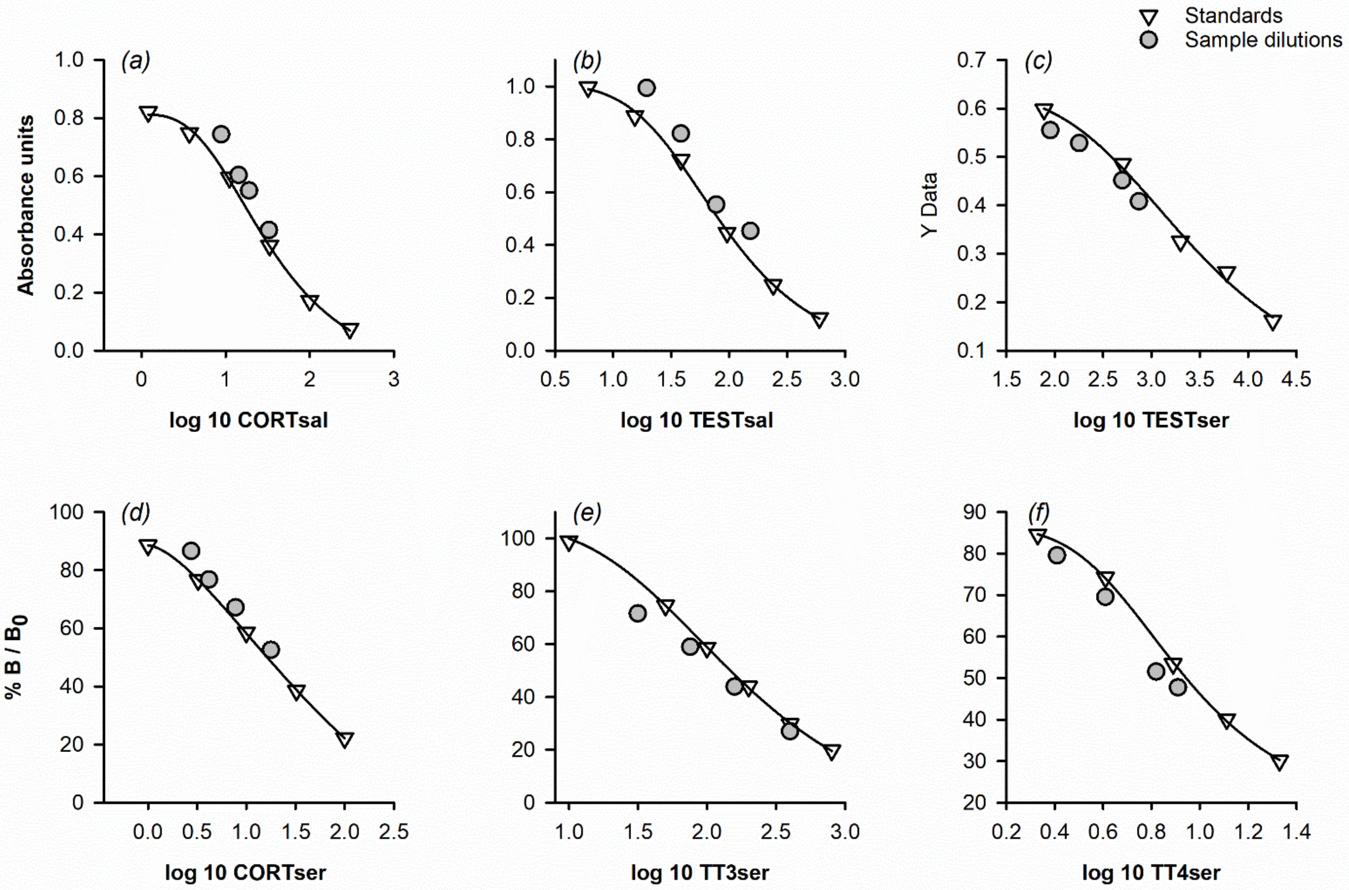

Supplementary Fig. 2 Salivary hormones (a) cortisol, $CORT_{sal}$, and (b) testosterone, $TEST_{sal}$, measured within individuals across two captures during the perinatal period. Fitted lines represents a 1:1 regression plot.

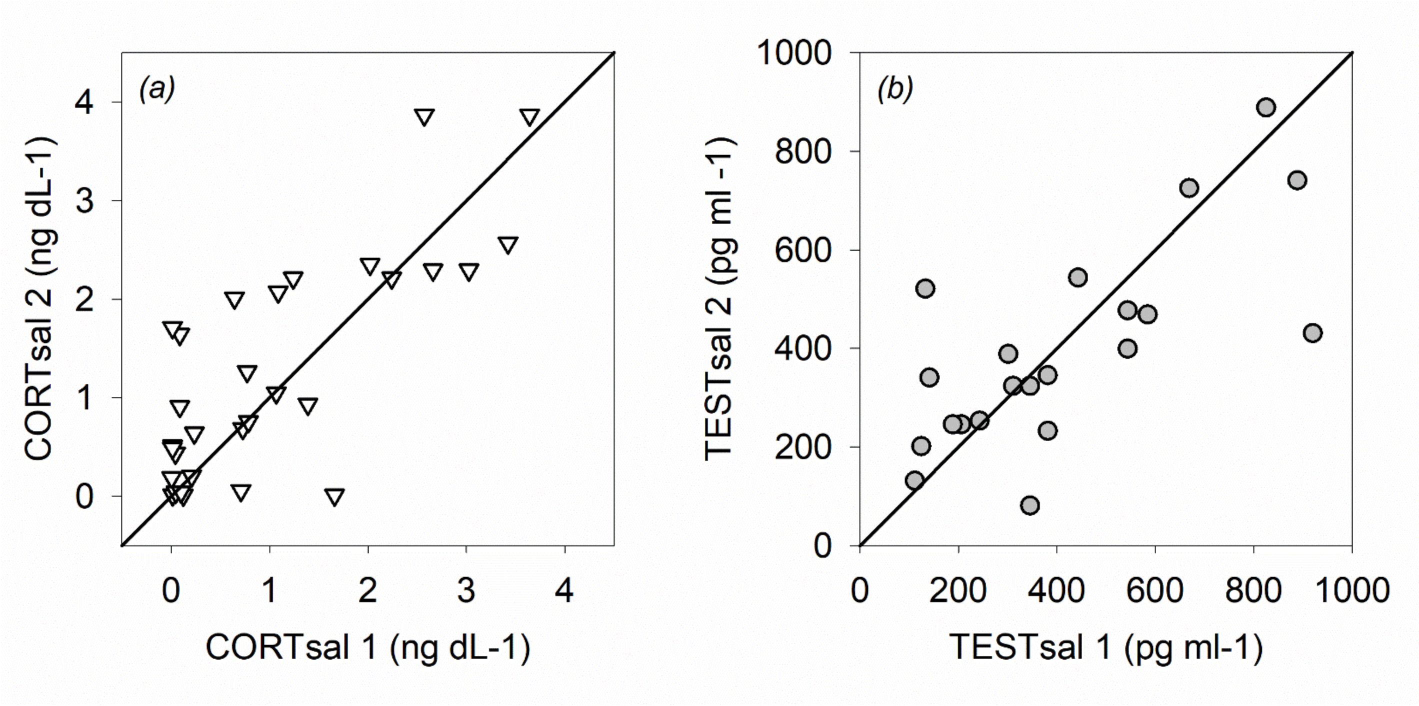

Supplement: Supplementary file 1 — Supplementary file1 (PDF 587 KB) [file 442_2020_4815_MOESM1_ESM.pdf]
